# AFF4 regulates osteogenic potential of human periodontal ligament stem cells via mTOR‐ULK1‐autophagy axis

**DOI:** 10.1111/cpr.13546

**Published:** 2023-09-20

**Authors:** Li Zhu, Jiahe Wang, Zuping Wu, Sirui Chen, Yuying He, Yukun Jiang, Guowen Luo, Zhuoxuan Wu, Yuyu Li, Jing Xie, Shujuan Zou, Chenchen Zhou

**Affiliations:** ^1^ State Key Laboratory of Oral Diseases, National Clinical Research Center for Oral Diseases, West China Hospital of Stomatology Sichuan University Chengdu China; ^2^ Stomatology Hospital, School of Stomatology, Zhejiang University School of Medicine, Zhejiang Provincial Clinical Research Center for Oral Diseases, Key Laboratory of Oral Biomedical Research of Zhejiang Province Cancer Center of Zhejiang University Hangzhou China; ^3^ Department of Orthodontics, West China Hospital of Stomatology Sichuan University Chengdu China; ^4^ Department of Pediatric Dentistry, West China Hospital of Stomatology Sichuan University Chengdu China

## Abstract

Scaffold protein AF4/FMR2 family member 4 (AFF4) has been found to play a role in osteogenic commitment of stem cells. However, function of AFF4 in human periodontal ligament stem cells (hPDLSCs) has not been studied yet. This present study aims to investigate the biological effect of AFF4 on osteogenic differentiation of hPDLSCs and potential mechanistic pathway. First, AFF4 expression profile was evaluated in conditions of periodontitis and osteogenic differentiation of hPDLSCs by immunohistochemical staining, western blot and qRT‐PCR. Next, si‐RNA mediated knockdown and lentiviral transduction mediated overexpression of AFF4 were adopted to explore impact of AFF4 on osteogenic capacity of hPDLSCs. Then, possible mechanistic pathway was identified. At last, pharmacological agonist of autophagy, rapamycin, was utilized to affirm the role of autophagy in AFF4‐regulated osteogenesis of hPDLSCs. First, AFF4 expressions were significantly lower in inflamed periodontal tissues and lipopolysaccharides‐treated hPDLSCs than controls, and were up‐regulated during osteogenic differentiation of hPDLSCs. Next, osteogenic potential of hPDLSCs was impaired by AFF4 knockdown and potentiated by AFF4 overexpression. Moreover, AFF4 was found to positively regulate autophagic activity in hPDLSCs. At last, rapamycin treatment was shown to be able to partly restore AFF4 knockdown‐suppressed osteogenic differentiation. Our study demonstrates that AFF4 regulates osteogenic potential of hPDLSCs via targeting autophagic activity. The involvement of AFF4 in periodontal homeostasis was identified for the first time.

## Graphical abstract

The present study shows that AFF4 protein expression was significantly lower in periodontitis tissues than healthy periodontal tissues. And we further revealed that AFF4 regulates osteogenic differentiation of hPDLSCs. The above findings provide a clue for possible engagement of AFF4 in local dyshomeostasis in periodontitis.

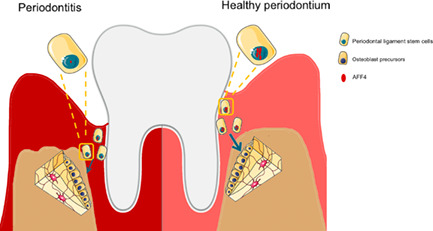

## 1 INTRODUCTION

Periodontitis has posed a significant health and financial burden due to substantial prevalence.^1^ This disease is induced by changed composition and amount of periodontal microbial populations, ultimately breaking host‐microbe homeostasis. The undifferentiated mesenchymal cells within PDL (PDL stem cells, hPDLSCs) are considered as ‘maintainers’ of periodontal homeostasis. hPDLSCs possess unique capability to fabricate the three‐dimensional structure of PDL tissues. Under specific signals or stimuli, hPDLSCs can differentiate into cementoblasts, osteoblasts and fibroblasts. Intriguingly, in alveolar bone niche, hPDLSCs have been proven to be a more robust source of osteogenic precursor cells compared to progenitors from periosteum and endosteum.^2^ Recovery of destroyed connective tissues and alveolar bone in periodontitis are tough based on current applicable therapeutics. In past two decades, exponential expanded studies have focused on hPDLSCs‐based cell therapy for periodontal regeneration, and these support its therapeutic potential in treatment of periodontal bone defects.^3^ Emerging evidence demonstrates that regenerative and differentiative capacities of hPDLSCs are affected in periodontitis microenvironment.^4^, ^5^, ^6^ Therefore, in‐depth explorations concerning critical regulators and underlying mechanistic network in the processes of osteogenic differentiation of hPDLSCs are still in demand.

Super elongation complex (SEC) is needed for rapid gene transcription via releasing RNA polymerase II at the pausing sites into productive transcription elongation.^7^ AF4/FMR2 family member 4 (AFF4), has been identified as the scaffolding protein of SEC. Its direct interaction with eleven‐nineteen Lys‐rich leukaemia (ELL) proteins, positive transcription elongation factor b (P‐TEFb) and frequent mixed lineage leukaemia (MLL) translocation partners are vitally important for SEC assembly. As the central factor of SEC, dysfunction of AFF4 contributes to both tumorigenesis and HIV transcription.^8^, ^9^, ^10^ Moreover, recent studies indicate that AFF4 also exerts effect in bone biology. Gain‐of‐function mutation of AFF4 has been reported to cause CHOPS syndrome, and one hallmark of which is skeletal development abnormality. Moreover, transcriptome analysis of those patients demonstrated that up‐regulated genes were enriched in skeletal system development/morphogenesis.^11^ Skeletal system derives from mesenchymal progenitor cells.^12^ Previous studies have identified the functions of AFF4 in lineage commitment of several types of mesenchymal stem cells. In human dental follicle cells, AFF4 was reported to regulate osteogenic differentiation of via manipulating ALKBH1, an important epigenetics regulator.^13^ In bone marrow derived mesenchymal stem cells (MSCs), osteogenic differentiation was repressed by AFF4 knockdown and enhanced by AFF4 overexpression. In vivo, mice with subcutaneously implanted AFF4‐overexpressing MSCs presented more new bone formation than control group. Regulatory action of AFF4 on osteogenic differentiation was found to be mediated by ID1 transcription, a downstream target of BMP2 signalling.^14^ As part of systemic skeletal system, periodontal alveolar bone possesses a unique mesenchymal progenitor and robust source of osteogenic lineages, PDLSCs. However, the function of AFF4 in hPDLSCs remains unclear yet. Therefore, revealing biological actions of AFF4 on osteogenesis of hPDLSCs will not only add to knowledge about how AFF4 regulates systemic bone metabolism, but also help enhance the understanding of periodontitis pathobiology.

Autophagy is responsible for eliminating damaged organelles, macromolecules and microbes in cells. It consists of multiple steps, including nucleation, elongation and maturation of autophagosome, and then fusion with lysosomes and degradation of enclosed materials. Autophagy has been found to participate in various physiological processes in mammals, including cellular homeostasis, immunity and inflammation, aging, tissue development and differentiation.^15^ The comprehensive impacts of autophagy in bone biology, osteogenesis in particular, have been widely accepted. Various exogenous and endogenous stimuli have been reported to modulate osteogenesis through autophagy regulation, including mechanical stimulation, hormones, and metabolic conditions.^16^ Autophagy also participate in the formation and mineralization of bone matrix.^17^ Previous work unveiled that AFF4 manipulates cellular differentiation of human mesenchymal stem cells, promoting adipogenic differentiation via regulating autophagy.^18^ But whether AFF4 regulates osteogenic differentiation of hPDLSCs, and the role of autophagy in this process awaits further exploration.

In this study, we performed AFF4 knockdown/overexpression to investigate the link between AFF4 and osteogenic capacity of hPDLSCs. According to RNA sequencing analysis, the impact of AFF4 on the basal level and flux of autophagy was further examined. At last, pharmacological autophagy activation was adopted to confirm the function of autophagy in AFF4‐regulated osteogenesis of hPDLSCs. And our data demonstrate that mTOR‐ULK1‐autophagy pathway underlying the regulatory actions of AFF4 on osteogenic differentiation of hPDLSCs.

## 2 MATERIALS AND METHODS

### 2.1 Sample collection

PDL tissues were obtained from populations referred to West China Hospital of Stomatology with informed consent and ethical approval (WCHSIRB‐D‐2023‐015). Tooth were included in periodontitis group when probing pocket depth (PPD) ≥ 4 mm and clinical attachment loss ≥ 4 mm are detected at 30% sites at least.^19^ PPD ≤ 2 mm and no bleeding on probing were considered as primary requirement for periodontally healthy tooth. After being scraped from tooth root, PDL tissues were washed with phosphate‐buffered saline (PBS) and fixed with 4% paraformaldehyde overnight at 4°C. Then, samples were dehydrated, embedment in paraffin before conducting serial tissue sections (5 μm).

### 2.2 Haematoxylin and eosin (H&E) staining and immunohistochemical staining

Paraffin slices were deparaffinized and rehydrated before H&E staining (HE; BL700A; Biosharp). Immunohistochemical staining was conducted with VECTASTAIN® Elite® ABC‐HRP Kit (PK‐6101; Vector laboratories). Slides were incubated in antigenic repair fluid for 20 min at 100°C for antigen retrieval. Endogenous peroxidase activity was quenched via incubation with 3% H_2_O_2_ for 30 min. Following PBS rinse, sections were further treated with goat serum for 1 h. Afterwards, incubation with corresponding primary antibodies were applied at 4°C overnight, including anti‐AFF4 (14662‐1‐AP; Proteintech), anti‐Collagen I (R26615; ZEN‐BIO), anti‐Runx2 (860139; ZEN‐BIO), and anti‐TNFα (346654; ZEN‐BIO). Thereafter, sections were treated with biotinylated goat anti‐rabbit IgG for 2 h and visualized with DAB peroxidase substrate kit (SK‐4100, Vector laboratories). Images were captured with light microscopy (Olympus).

### 2.3 Cell cultivation, phenotypic characterization and osteogenic differentiation induction

hPDLSCs in this study were isolated and cultivated according to protocols published previously.^20^ hPDLSCs were derived from periodontally healthy third molars and premolars of systemically healthy patients aged between 12 and 25 years at West China Hospital of Stomatology. After rinsing with sterile PBS, the middle third PDL tissues on root were scraped and cultivated in growth medium, namely 1% penicillin–streptomycin and 10% foetal bovine serum (FBS; Sigma–Aldrich) supplemented α‐minimum essential medium (α‐MEM; Gibco). Cells between passage 2 and 5 were used in this investigation.

Phenotypic characterization of hPDLSCs was conducted through immunofluorescent staining and multipotential differentiation induction. Osteogenic differentiation was inducted with growth medium supplemented with 50 μg/mL β‐ascorbic acid (J&K), 20 nM dexamethasone (Sigma–Aldrich), and 8 mM β‐glycerol phosphate (Sigma–Aldrich). For qRT‐PCR and western blotting, induction of 3 days was adopted.

### 2.4 si‐RNA interference and lentiviral transduction

For small interfering (si)‐RNA transfection, hPDLSCs were plated and cultured in growth medium without antibiotics. Then hPDLSCs were subjected to si‐RNA (100 nM) transfection with Lipofectamine RNAiMAX (Invitrogen) for 6 h. After replacing with growth medium without antibiotics, hPDLSCs were cultured for 48 h before osteogenic differentiation induction. si‐RNA plasmids targeting AFF4 and negative control siRNA (siControl) were constructed by Huzhou Hippo Biotechnology Co., Ltd. The silencing efficiency was affirmed by qRT‐PCR at 48, 60 and 72 h.

Lentivirus for AFF4 overexpression (oeAFF4) and control vector were provided by Shanghai Genechem Co., Ltd. Lentiviral transfection was performed upon 30% cell confluence for 24 h before replacing with fresh growth medium. Transfection efficiency was verified by western blot analysis and qRT‐PCR at 72 h.

### 2.5 RNA extract and real‐time quantitative reverse transcription PCR (qRT‐PCR)

Total RNA was obtained with the Pure RNA Isolation Kit (RP5611; Bioteke Corporation), and quantification of which was conducted with Nano Spectrophotometer 2000c (Thermo Fisher Scientific). cDNA synthesis was performed with RevertAid First Strand cDNA Synthesis Kit (K1622; Thermo Fisher Scientific). Real‐time amplification was detected with ChamQ SYBR Color qPCR Master Mix (Q411‐02; Vazyme) on the iCycler (Bio‐Rad) according to previous report.^21^ *GAPDH* was used for normalization, and relative quantities of target genes were evaluate with the 2^−ΔΔCt^ method. Primer sequences used in this experiment are provided in Table S1.

### 2.6 Protein extract and western blot analysis

Protein contents were extracted with RIPA lysis buffer (P0013B; Beyotime) supplemented with 1% proteinase inhibitor phenyl‐methylsulfonyl fluoride (PMSF; ST506‐2; Beyotime). Isolated target protein on SDS‐PAGE gels were transferred onto PVDF membranes (IPVH00010; Millipore). The blotting membranes were treated with 5% skim milk for 1 h and then with primary antibody overnight (12–16 h). Thereafter, blotting bands were incubated with secondary antibodies at room temperature for 2 h before visualization with Immobilon® Western (P90719, Millipore). In this study, primary antibodies (1:1000) included: anti‐β‐actin (200068‐8F10; ZEN‐BIO), anti‐AFF4 (14662‐1‐AP; Proteintech), anti‐LC3B (18725‐1‐AP; Proteintech), anti‐Collagen I (R26615; ZEN‐BIO), anti‐Runx2 (860139; ZEN‐BIO), anti‐ALP (381009; ZEN‐BIO), anti‐ULK1(381887; ZEN‐BIO), anti‐Phospho‐ULK1 (Ser758) (AF4387; Affinity Biosciences), anti‐mTOR (380411; ZEN‐BIO), anti‐Phospho‐mTOR (Ser2448) (381557; ZEN‐BIO). Secondary antibodies included: Goat anti‐mouse IgG (H&L) (HRP conjugated) (511103; ZEN‐BIO) and Goat anti‐rabbit IgG (H&L) (HRP conjugated) (511203, ZEN‐BIO).

### 2.7 Alkaline phosphatase (ALP) and alizarin red (ARS) staining

Cells were cultured in osteogenic medium for 7 days before ALP staining, and for 14 days before ARS staining. After PBS washing, 4% paraformaldehyde was added to fix cells for 0.5 h. Then. PBS rinse was required again to washing off paraformaldehyde residual. BCIP/NBT ALP Color Development kit (C3206; Beyotime) was used for ALP staining, and 1% Alizarin Red S solution (G1452; Solarbio) was applied to staining the calcified nodules according to manufacturers' instruction. A light microscope was used to collect stained images (Olympus).

### 2.8 Immunofluorescent staining

hPDLSCs were seeded on confocal cell culture dishes for immunofluorescent staining. First, 4% paraformaldehyde was utilized for cell fixation of harvested samples for 10 min. Second, cells were subjected to Triton X‐100 (0.25%; Beyotime) for cell permeabilization for 10 min, and to 5% BSA for blocking for 1 h at room temperature. Subsequently, cells were firstly incubated with primary antibodies dilutions of AFF4 (1:300), LC3B (1:300) at 4°C overnight, and then with Donkey Anti‐Rabbit IgG H&L (Alexa Fluor® 647) (ab150075; Abcam) for 2 h at room temperature. 4–6‐diamidino‐2‐phenylindole (DAPI; D9542; Sigma–Aldrich) were used to stain nuclei. Finally, we captured immunofluorescent images under CLSM (FV3000, Olympus, Japan).

### 2.9 Transmission electron microscope

hPDLSCs were incubated in 60 mm cell dishes and treated with si‐RNA according to protocols. Cells were washed, trypsinized and centrifuged, then cells sediments were fixed with 3% glutaraldehyde firstly and with 1% osmium tetroxide (OsO4) finally. Transmission electron microscope (JEM‐1400FLASH) was used to detect autophagosomes in the fixed cells at Lilai biomedicine experiment centre (Chengdu, China).

### 2.10 RNA sequencing

hPDLSCs were plated on 6 well plates followed by si‐RNA interference for 48 h. Cell lysates were collected with Trizol (No. 15596‐026, Thermo Fisher Scientific), and sent to Seqhealth Technology Co., LTD (Wuhan, China) for UID RNA‐seq experiment and high through‐put sequencing, as well as data analysis.

### 2.11 Statistical analysis

Experiments were conducted in triplicate independently. Quantitative data are displayed as the mean ± standard deviation (S.D.). Unpaired *t*‐test and ANOVA was adopted for comparison between two groups or for multiple comparisons. The *p* value less than 0.05 was defined as statistically significant.

## 3 RESULTS

### 3.1 AFF4 level correlates with periodontal osteogenic activity in inflamed and healthy conditions

**FIGURE 1:**
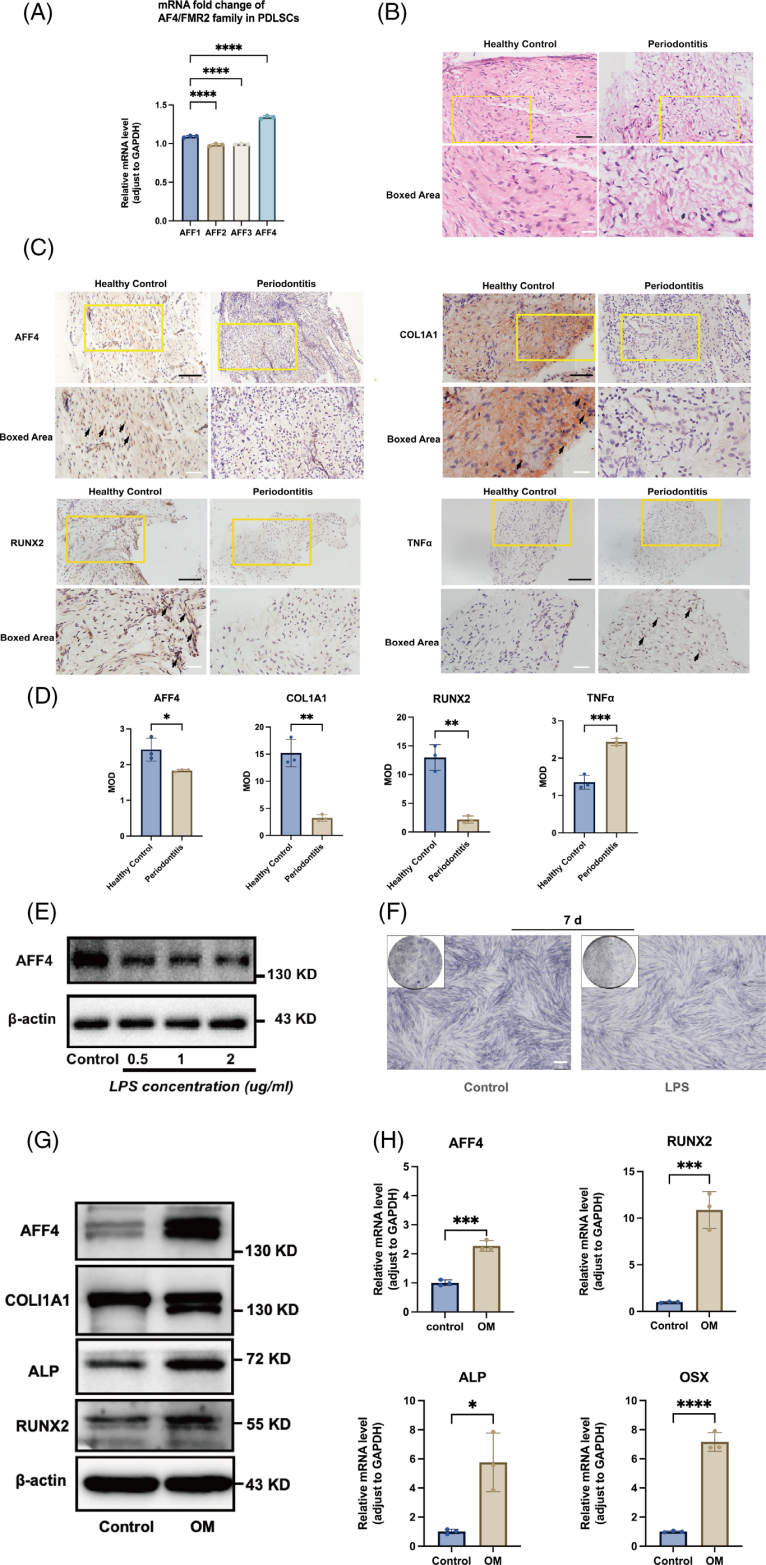
AFF4 level correlates with periodontal osteogenic activity in inflamed and healthy conditions. (A) Quantification of AF4/FMR2 family expression at mRNA level via qRT‐PCR. *GAPDH* is served as internal control. The data are shown as the means ± SD. Data were based on three independent experiments (*n* = 3); ****p < 0.0001. PDL tissues of periodontitis and periodontally healthy populations were treated with H&E and immunohistochemical staining. (B) Representative images of H&E‐stained section were shown. Black scale bar, 50 μm; white scale bar, 20 μm. (C) Representative images and (D) the means of integrated optical density (MOD) analysis of IHC staining of AFF4, COL1A1, RUNX2 and TNFα in PDL tissues. Immunohistochemical positive areas are marked by black arrow. Black scale bar, 100 μm; white scale bar, 50 μm. *n* = 3; *p < 0.05; **p < 0.01; ***p < 0.001. (E) Western blot identified LPS‐induced AFF4 expression change in hPDLSCs. (F) ALP staining images (7 d) demonstrate LPS‐induced compromised osteogenesis of hPDLSCs. LPS concentration, 1 μg/mL. Scale bar, 500 μm. hPDLSCs were subjected to osteogenic induction for 3 days, and (G) up‐regulated protein levels of AFF4, RUNX2, ALP and COL1A1 were identified by western blot. (H) qRT‐PCR analysis quantified relative gene expression of *AFF4*, *RUNX2*, *ALP* and *OSX* normalized to GAPDH. Values are shown as the means ± SD. *n* = 3; *p < 0.05; ***p < 0.001; ****p < 0.0001. OM, osteogenic mineralization induction.

The regulatory roles of AF4/FMR2 family members in osteogenic potential of human MSCs and human dental follicle cells have been preliminarily revealed.^13^, ^14^ However, the biological functions of this family in hPDLSCs have yet to be investigated. In present study, qRT‐PCR analysis assessed innate expression profile of AF4/FMR2 family in hPDLSCs, and showed that AFF4 exhibited highest expression level (Figure 1A). Given that the osteogenic differential potential of the periodontium has been reported to be affected by periodontal inflammation.^4^, ^5^, ^6^, ^20^ We applied histological techniques to detect AFF4 expression and distribution in periodontal tissues. As shown by H&E staining, connective tissues in the periodontitis group were swollen, with disorganized fibres and dilated blood vessels, compared to the healthy control (Figure 1B). Immunohistochemical staining demonstrated that the periodontitis group showed increased expression of the inflammatory marker (TNFα), along with decreased levels of AFF4 and osteogenic protein (RUNX2, COL1A1) (Figure 1C,D). In addition, lipopolysaccharides (LPS) treatment for 24 h led to down‐expression of AFF4 in a concentration‐dependent manner (Figure 1E). LPS (1 μg/mL) treatment did not significantly impact cell proliferative activity (Figure S4). Combined LPS (1 μg/mL) and osteogenic medium treatment induced less ALP expression than control cells with osteogenic induction only (Figure 1F). To further confirm the correlation between AFF4 and osteogenic activity of hPDLSCs, hPDLSCs were isolated and phenotypically identified. Immunofluorescent staining identified the expression of the MSC surface marker CD90 on these cells (Figure S1C). Furthermore, adipogenic, chondrogenic, and osteogenic differentiation capacities were all detected (Figure S1D–F). After osteogenic induction of hPDLSCs for 3 days, both western blot analysis and qRT‐PCR results indicated obvious increase of AFF4 expression during osteogenic differentiation of hPDLSCs (Figure 1G,H). These results implicate involvement of AFF4 in osteogenic differentiation of hPDLSCs in both health and disease states.

### 3.2 Osteogenic capacity of hPDLSCs is compromised by AFF4 knockdown and potentiated by AFF4 overexpression

**FIGURE 2:**
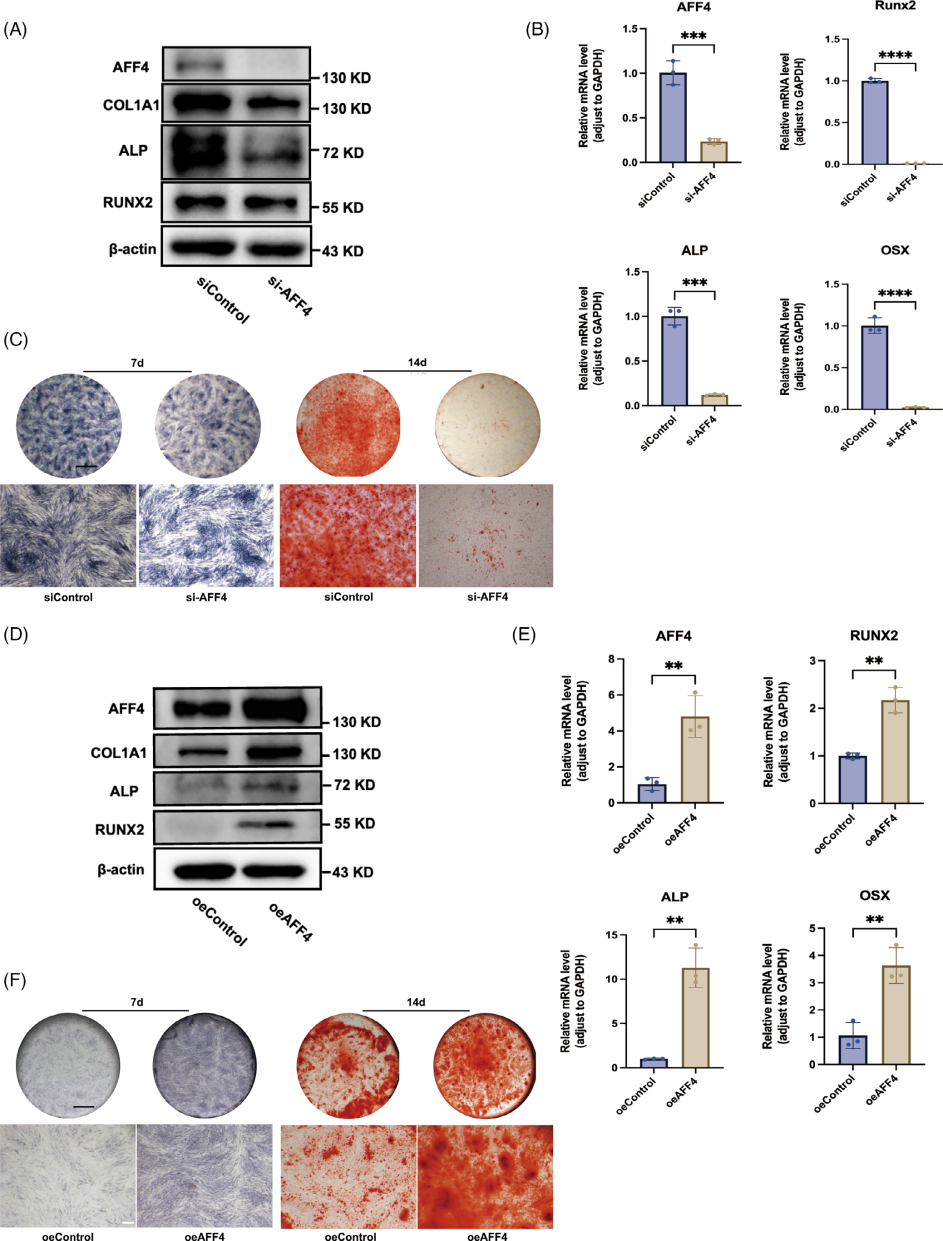
Osteogenic capacity of PDLSCs is compromised by AFF4 knockdown and potentiated by AFF4 overexpression. hPDLSCs were subjected to osteogenic induction after transfection with siRNA targeting AFF4 or negative control siRNA for 48 h. (A) Western blot analysis (3 days) of AFF4 knockdown and expression of osteogenic proteins (RUNX2, ALP, COL1A1). (B) qRT‐PCR analysis (3 days) of gene expressions of *AFF4* and osteogenic genes (*RUNX2, ALP, OSX*). Values are displayed as the means ± SD. *n* = 3; ***p < 0.001; ****p < 0.0001. (C) ALP staining at 7 days (left) and ARS staining at 14 days (right) were carried out to visualize alkaline phosphatase activity and mineralized nodules respectively. Black scale bar, 2 mm; white scale bar, 500 μm. hPDLSCs were transected with lentivirus for AFF4 overexpression, and followed by osteogenic induction. (D) Efficiency of AFF4 overexpression and expression of osteogenesis‐related proteins (RUNX2, ALP, COL1A1) were assessed by western blot (3 days). (E) Quantitative measurement of *AFF4*, *RUNX2*, *ALP* and *OSX* gene expression were performed by qRT‐PCR (3 days). Values are displayed as the means ± SD. *n* = 3; **p < 0.01. (F) Images of ALP staining at 7 days (left) and ARS staining at 14 days (right) were shown. Black scale bar, 2 mm; white scale bar, 500 μm.

To further investigate the effect of AFF4 on the osteogenic capacity of hPDLSCs, si‐RNA interference technique was applied to deplete AFF4 in hPDLSCs. Knockdown efficiency was assessed at 48 h, 60 h and 72 h by qRT‐PCR (Figure S2), and 48 h‐transfection was adopted in subsequent experiments. hPDLSCs were treated with siRNA for 48 h initially, and then subjected to osteogenic induction for 3 days for western blot analysis and qRT‐PCR. For ALP and ARS staining, osteogenic differentiation induction was applied for 7 days and 14 days respectively. The qRT‐PCR data showed that AFF4 knockdown inhibits the expression of osteogenic mRNAs, including *RUNX2*, *ALP* and *OSX* (Figure 2B). Western blot analysis further confirmed this effect, evidenced by decreased protein expressions of RUNX2, ALP and COL1A1 in si‐AFF4 group compared to group treated with negative control si‐RNA (Figure 2A). ALP staining revealed reduced alkaline phosphatase (an enzyme related with early osteogenesis) and ARS staining showed fewer mineralized nodules in response to AFF4 silencing (Figure 2C).

Next, overexpression effect of a lentivirus‐based AFF4 expression system was evaluated by both western blot analysis and qRT‐PCR (Figure 2D,E). AFF4 overexpression was found to enhance the osteogenic differentiation of hPDLSCs. Both qRT‐PCR and western blot analysis detected up‐regulated expression of osteogenic markers (RUNX2, ALP, COL1A1 and OSX) (Figure 2D,E). Alkaline phosphatase and calcium nodules were increased in AFF4 overexpression group compared to vector‐treated controls (Figure 2F). These results confirm that AFF4 positively regulates osteogenic differentiation of hPDLSCs.

### 3.3 AFF4 knockdown induced differential expression of autophagy‐related pathway in hPDLSCs

**FIGURE 3:**
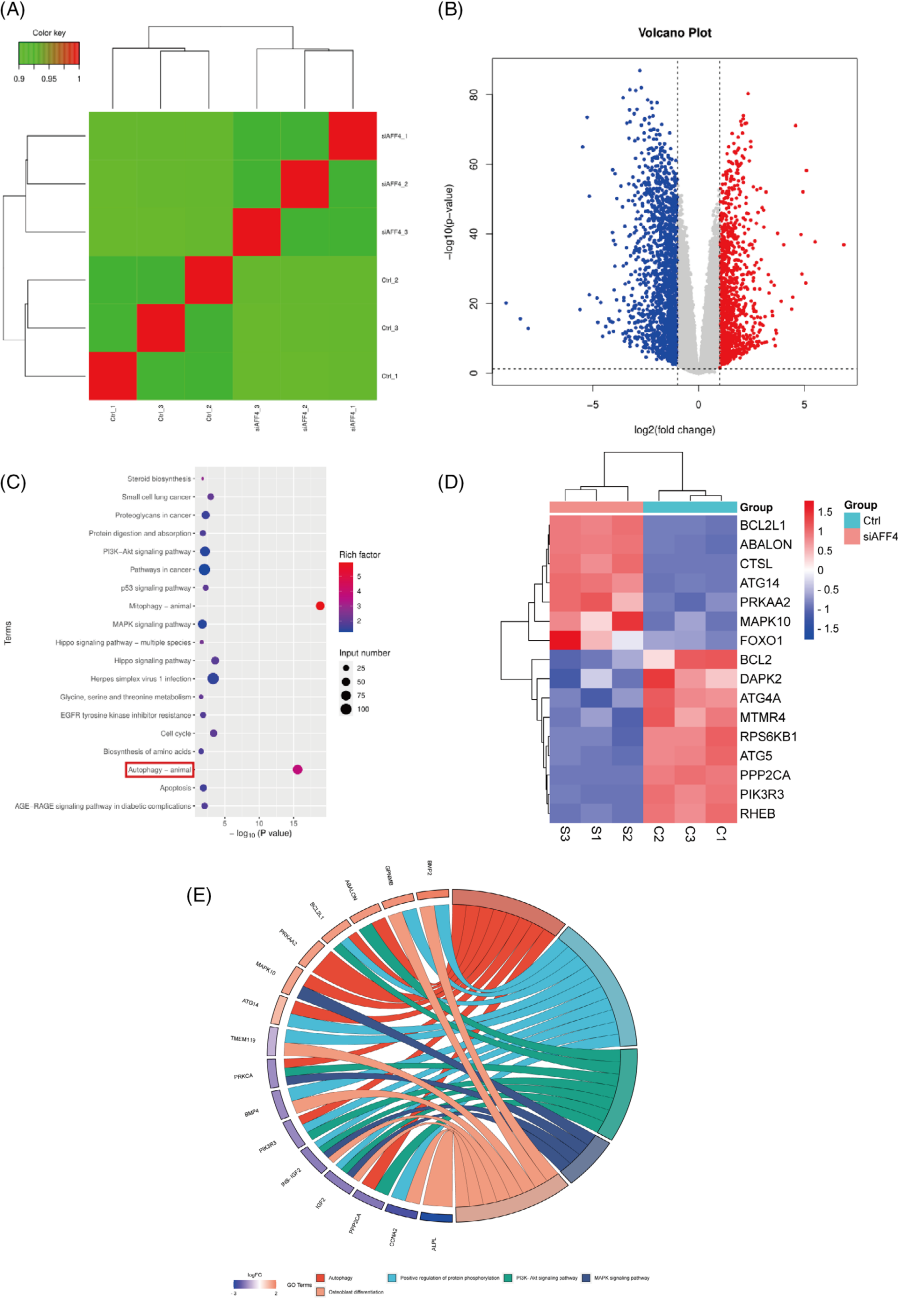
AFF4 knockdown induced differential expression of autophagy‐related pathway in hPDLSCs. RNA‐seq data for AFF4 knockdown were based on statistical analysis of three independent samples. (A) Correlation heatmap showing the relationship between samples in si‐AFF4 and siControl hPDLSCs. (B) Volcano plot displaying differentially expressed genes. (C) KEGG pathway enrichment analysis for differentially expressed genes. (D) Heatmap of autophagy‐related genes. (E) Chord diagram showing possible cross‐talk between enriched signalling pathways.

To explore the underlying mechanism, RNA sequencing analysis was conducted to identify differential expression of gene profile induced by AFF4 knockdown in hPDLSCs. The RNA‐seq results showed an acceptable level of repeatability within groups (Figure 3A). si‐RNA‐mediated AFF4 depletion altered the expressions of 2925 genes, among which 1251 genes were up‐regulated and 1674 genes were down‐regulated (Figure 3B). Moreover, remarkably varied expressions of autophagy‐related genes were detected (Figure 3D), which corroborated previous studies.^18^ GO term and KEGG enrichment analysis further verified the down‐regulation of autophagy‐associated pathways and osteoblast differentiation (Figure 3C,E).

### 3.4 AFF4 modulates autophagy activity in hPDLSCs

**FIGURE 4:**
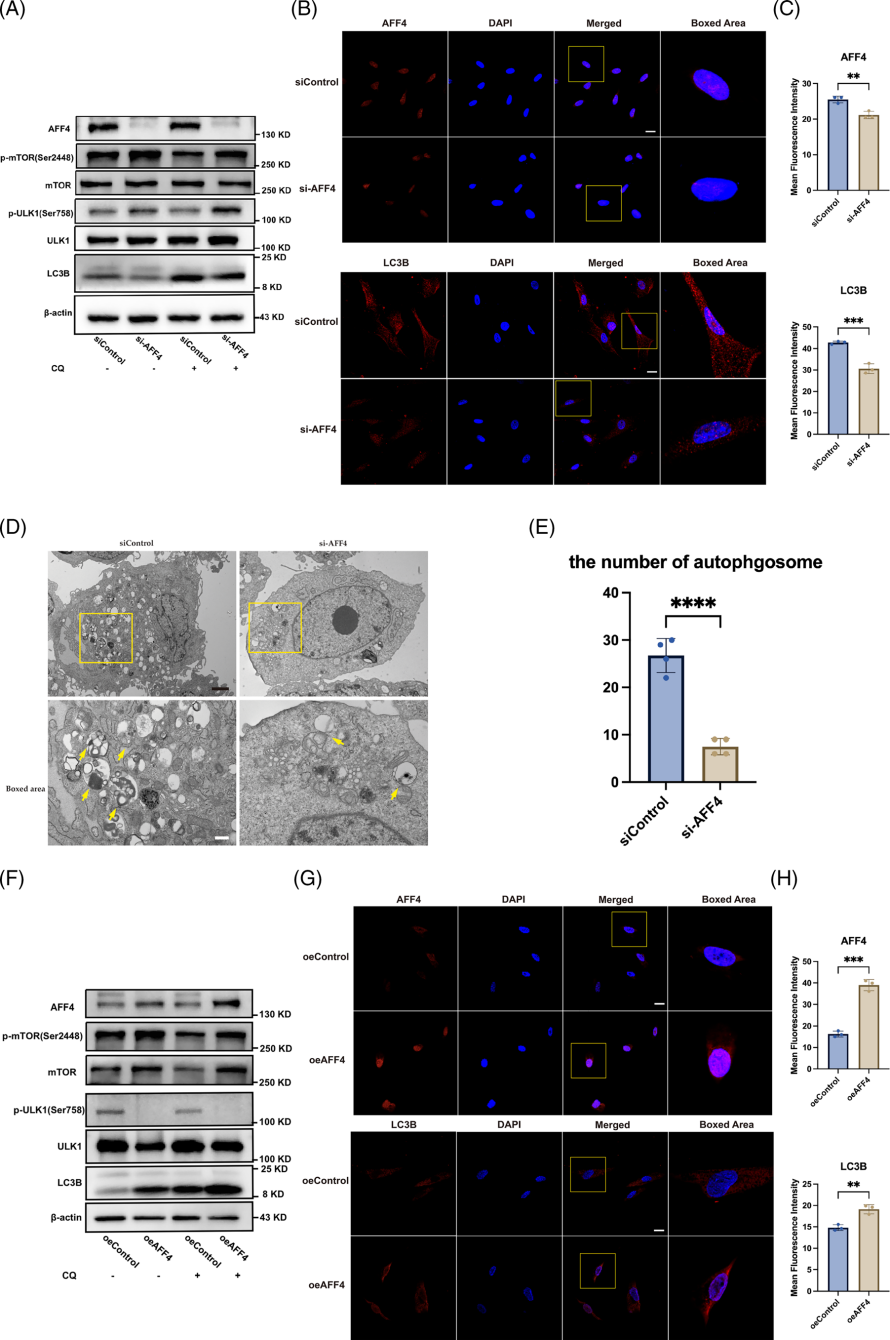
AFF4 modulates autophagy activity in hPDLSCs. Cells were treated with CQ (chloroquine, 40 μM) for 2 h before harvest to measure autophagy afflux. (A) Western blot showing autophagy specific markers, p‐mTOR, mTOR, p‐ULK1, ULK1 and LC3B in siControl and si‐AFF4 hPDLSCs. (B) Representative immunofluorescent images and (C) statistical analysis for mean fluorescent intensity of AFF4 and LC3B puncta in siControl and si‐AFF4 hPDLSCs. Scale bar, 20 μm. *n* = 3; **p < 0.01; ***p < 0.001. (D) Representative images and (E) quantification of autophagic vacuoles in siControl and si‐AFF4 hPDLSCs. Autophagosome and autolysosome‐like structures were marked by yellow arrow. Black scale bar, 2 μm; White black bar, 500 nm. Data are presented as the means ± SD. *n* = 4; ****p < 0.0001. (F) Expression of autophagy‐related proteins, p‐mTOR, mTOR, p‐ULK1, ULK1 and LC3B were assessed in oeAFF4 and oeControl hPDLSCs. (G) Representative immunofluorescent images and (H) histograms for mean fluorescent intensity of AFF4 and LC3B puncta in oeControl and oeAFF4 hPDLSCs. Scale bar, 20 μm. *n* = 3; **p < 0.01; ***p < 0.001.

To confirm high‐throughput sequencing data, we further detected autophagy activity in hPDLSCs in conditions of AFF4 depletion and overexpression. Western blot, immunofluorescence, and TEM analysis were carried out to monitor autophagy level. Additionally, CQ treatment (40 μM) was applied 2 h before harvest to measure autophagic flux. Western blot indicated that AFF4 depletion enhanced phosphorylation of mTOR (Ser2448) and ULK1 (Ser758), and reduced protein expression of LC3B II/I. With combined CQ treatment, the level of LC3B II remained lower in si‐AFF4 group relative to siControl (Figure 4A). Immunofluorescent analysis displayed reduction of LC3B puncta induced by AFF4 depletion (Figure 4B,C). TEM also showed significantly fewer autophagic vacuoles in si‐AFF4 group than controls (Figure 4D,E). Correspondingly, AFF4 overexpression increased protein expression of LC3B II/I, while reduced the relative protein amount of p‐mTOR (Ser2448)/mTOR and p‐ULK1 (Ser758)/ULK1 (Figure 4F). Obviously increased immunofluorescent puncta of LC3B induced by AFF4 overexpression further confirmed results of western blot (Figure 4G,H).

### 3.5 Autophagy activation partially rescued osteogenic potential of hPDLSCs compromised by AFF4 knockdown

**FIGURE 5:**
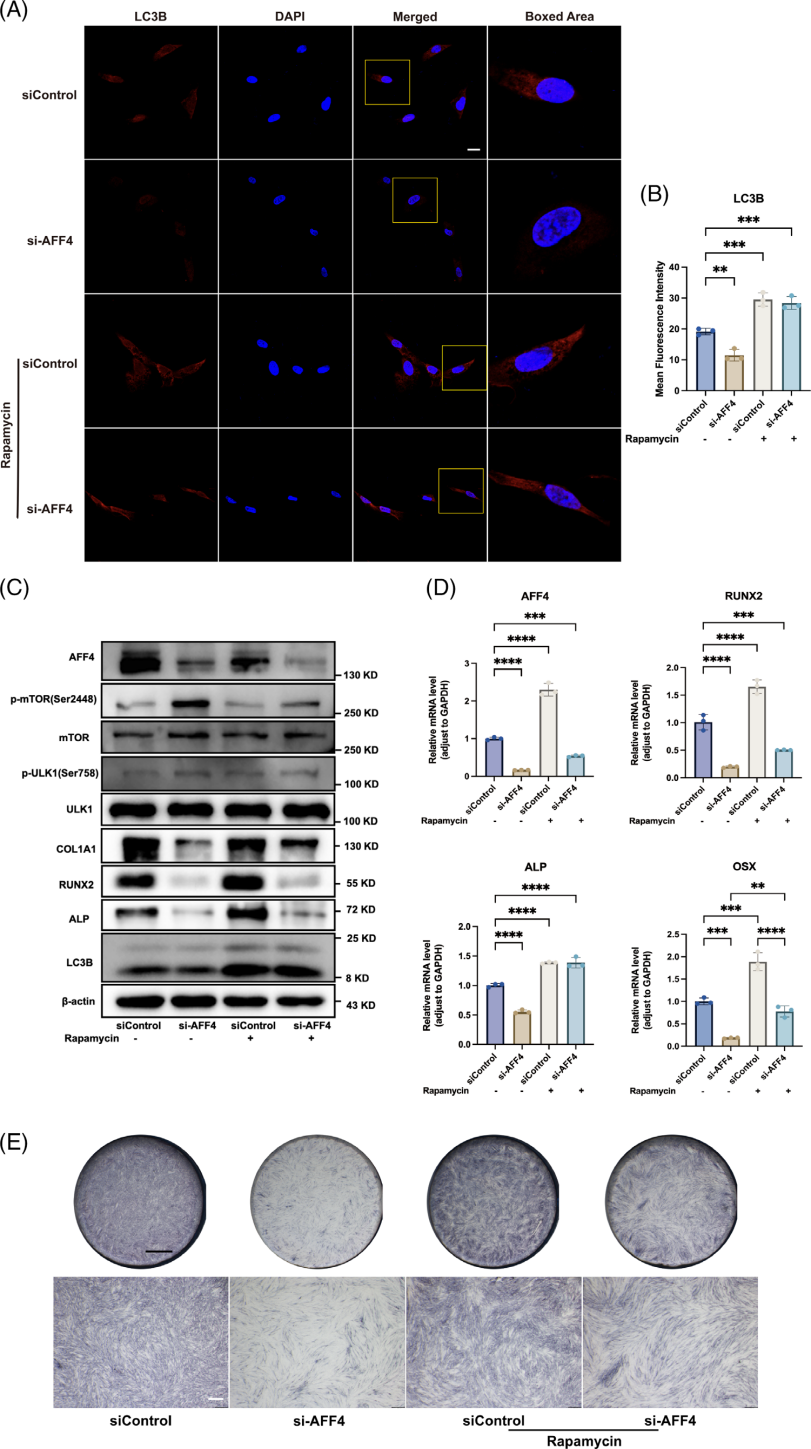
Autophagy activation partially rescued osteogenic potential of hPDLSCs compromised by AFF4 knockdown. (A) Representative immunofluorescent staining showing intensity of LC3B puncta in condition of AFF4 knockdown and rapamycin (100 nM) treatment. Scale bar, 20 μm. (B) Quantification for mean fluorescent intensity of LC3B puncta. *n* = 3; **p < 0.01; ***p < 0.001. (C) Western blot showed that rapamycin treatment (100 nM) increased autophagy‐related proteins (p‐mTOR, mTOR, p‐ULK1, ULK1, LC3B), and osteogenic markers (RUNX2, ALP, COL1A1) at 3 days. (D) Osteogenic genes expression at 3 days (*RUNX2*, *ALP*, *OSX*) were quantified by qRT‐PCR. Values are displayed as the means ± SD. *n* = 3; **p < 0.01; ***p < 0.001; ****p < 0.0001. (E) ALP staining at 7 days was performed. Black scale bar, 2 mm; white scale bar, 500 μm.

Finally, based on the RNA‐seq results, we endeavoured to validate the role of autophagy in osteogenesis regulated by AFF4 in hPDLSCs. Rapamycin, an inhibitor of mTORC1, was utilized to pharmaceutically activate autophagy. A 4 h treatment with rapamycin (100 nM) significantly enhanced autophagy activity in hPDLSCs, as identified by an increase in LC3B puncta under CLSM (Figure 5A,B). Moreover, osteogenesis compromised by AFF4 knockdown in hPDLSCs was partially rescued by rapamycin. After siRNA transfection, hPDLSCs were subjected to osteogenic induction with or without simultaneous rapamycin stimulation. Western blot analysis demonstrated that rapamycin stimulation significantly enhanced protein expressions of ALP and COL1A1, while slightly for RUNX2 (Figure 5C). Meanwhile, the downtrend of osteogenic mRNA expression (*RUNX2*, *ALP*, *OSX*) was reversed by rapamycin (Figure 5D). ALP staining showed that rapamycin increased alkaline phosphatase induced by AFF4 knockdown (Figure 5E).

## 4 DISCUSSION

Chronic periodontitis is characterized by loss of connective tissue attachment and alveolar bone. Moreover, the regenerative potential of resident mesenchymal stem cells, primarily hPDLSCs, were discovered to be compromised in inflammatory condition.^4^, ^5^, ^6^, ^20^ Determining pivotal factors that manipulate osteogenic commitment of hPDLSCs in periodontitis niche is in urgent demand. AFF4, the SEC scaffold protein, has been gradually recognized for its function in skeletal system in the past decade. Previous studies of our lab have revealed that AFF4 regulates osteogenic potential of human mesenchymal stem cells and human dental follicle cells.^13^, ^14^ However, the function of AFF4 in osteogenic differentiation of hPDLSCs remains an enigma yet.

At the beginning of our study, we identified that AFF4 is the dominant AF4/FMR2 family member in hPDLSCs. Then we detected periodontal AFF4 expression in both inflamed and healthy conditions. On the one hand, immunohistochemical analysis of PDL tissues revealed that AFF4 proteins were down‐expressed in periodontitis populations, in accordant with osteogenic proteins. Moreover, LPS was applied to hPDLSCs to mimic inflammatory state in vitro. LPS treatment decreased AFF4 expression and impaired osteogenic differentiation of hPDLSCs. On the other hand, up‐expression of AFF4 during osteogenic differentiation was also observed. Consistent alteration of AFF4 and osteogenic markers suggests that AFF4 might act as an important regulatory molecule for osteogenesis in condition of periodontitis. Before testing this hypothesis, the effect of AFF4 on osteogenesis of hPDLSCs in physiological conditions should be determined, which is also the primary concern of present study. We adopted siRNA interference and lentiviral transfection techniques to manipulate AFF4 expression in hPDLSCs. We observed that knockdown of AFF4 inhibited osteogenesis of hPDLSCs via western blot, qRT‐PCR, ALP and ARS staining. Meanwhile, AFF4 overexpression was confirmed to elevate expression of osteogenic mRNAs and proteins (RUNX2, ALP, COL1A1, OSX), as well as alkaline phosphatase and calcified nodules. Based on our study results, regulatory role of AFF4 in osteogenic commitment of hPDLSCs is established for the first time. And our finding is in accordance with similar studies in other stem cells.^13^, ^14^

Critical role of autophagy in osteogenic commitment of mesenchymal stem cells has been substantially verified. Autophagy has been found to positively regulate osteogenic differentiation of bone marrow BMCs (BMMSCs) and MSCs originated from human gingiva (HGMSC).^21^, ^22^, ^23^ Decreased autophagic activity controls development of osteoporosis, and autophagy activation could recue osteogenic differentiation of BMMSCs and alleviate osteoporotic phenotype in ovariectomy mice model and aged mice.^21^, ^22^ Osteogenic differentiation of human MSCs is companied by shifted cellular metabolic patterns and increased endogenous reactive oxygen species (ROS). Autophagic activity was found to be essential for osteogenic differentiation of hMSCs, acting as critical antioxidant mechanism for ROS clearance.^24^ Highly accumulated autophagic vacuoles were observed in undifferentiated BMMSCs, which undergo rapid degradation upon induction of osteogenesis. It is suggested that these accumulated autophagosomes may be responsible for rapid generation of energy substrates that support the osteogenesis of MSCs.^25^ In periodontal milieu, impaired autophagosome‐lysosome fusion and dysfunctional lysosomes were found to contribute to compromised osteogenesis of hPDLSCs under inflammatory condition.^26^ Autophagy has also been identified to enhance osteogenesis of hPDLSCs in vitro and increase newly‐formed alveolar bone in vivo in response to treatment with various gold nanoparticles biomaterials.^27^, ^28^, ^29^ Moreover, autophagy activities has been found to mediate the regulatory function of AFF4 on lineage commitment of hMSCs.^18^ In this study, we carried out RNA sequencing analysis and found that AFF4 knockdown caused significant enrichment of autophagy‐related pathways in hPDLSCs, corroborating finding of the previous study. Both aforementioned findings and transcriptome analysis hint that changed autophagic activities possibly involve in AFF4‐regulated osteogenic differentiation of hPDLSCs. Interestingly, mitophagy‐related pathways also exhibited significant enrichment after AFF4 knockdown, which is expected to be further explored in future researches.

mTOR (mammalian target of rapamycin) is a critical autophagy regulator, coordinating autophagy with cellular metabolism to maintain cell homeostasis. mTORC1 is the primary functional complex of mTOR in regulating autophagy. mTORC1 inhibits autophagy via phosphorylating various autophagy‐related proteins which are essential for initiation and nucleation of autophagosomes.^30^ ULK1 complex, formed by ULK1/2, mAtg13 and FIP200, is essential for autophagy initiation. mTOR suppresses ULK1 activation by phosphorylating ULK1 Ser758 (human) and thus inhibiting autophagy activation.^31^ Contribution of mTOR signalling in regulating osteogenesis of stem cells has been substantially verified.^32^, ^33^, ^34^ Among mTOR‐related pathways, mTOR/ULK1 autophagic axis has been identified to mediate autophagy activation and osteogenic commitment of BMSCs.^35^, ^36^ Therefore, it is hypothesized that mTOR‐ULK1‐autophagy pathway engages in AFF4‐regulated osteogenic commitment of hPDLSCs. Atg8‐family proteins are essential for autophagosome biogenesis via conjugating to phosphatidylethanolamine on autophagic membrane to initiate phagophore elongation. And MAP1LC3B/LC3B has been extensively studied and accepted as an excellent autophagic protein marker among mammalian homologues of Atg8‐family proteins.^37^ LC3B includes LC3B I and LC3B II (the PE‐conjugated form of LC3B‐I), and the latter localizes on autophagosomal membrane. Therefore, it is more reliable to examine conversion rate of LC3B I to LC3B II rather than the amount of LC3B proteins alone. In present study, phosphorylation level of mTOR (Ser2448) and ULK1(Ser758), along with LC3B II/I expression were examined to reflect autophagy activity. Considering that autophagosomes are in constant formation and degradation, both basal autophagy level and autophagy flux were monitored in our study. Phosphorylation of mTOR (Ser2448) and ULK1(Ser758) were enhanced in response to AFF4 knockdown, and suppressed by AFF4 overexpression. Additionally, LC3B II/I was down‐expressed in si‐AFF4 group and up‐expressed in oeAFF4 group at protein level. Although LC3B level was increased in both si‐AFF4/CQ and siControl/CQ group compared to groups without CQ treatment. Lower LC3B expression in si‐AFF4/CQ than in siControl/CQ group excluded the possibility of misinterpreting increased autophagy flux as autophagy inhibition. The results of AFF4 overexpression with combined CQ treatment also verified this fact. LC3B expressions were further confirmed by immunofluorescence. Besides, TEM identified that autophagic vacuoles in si‐AFF4 group is significantly decreased, fewer than half of those in siControl groups (*p* < 0.0001). In conclusion, basal autophagy activity and autophagy flux were suppressed by AFF4 knockdown, and enhanced by AFF4 overexpression. These results corroborate RNA‐seq data, as well as our previous report that AFF4 positively regulates autophagy in human mesenchymal stem cells and favouring their adipogenic differentiation.^18^

To further verify the role of autophagy in the relationship between AFF4 and osteogenesis of hPDLSCs, rapamycin was applied to recue AFF4 knockdown‐induced autophagy inhibition in hPDLSCs. Autophagy activation was evidenced by reduced phosphorylation of ULK1(Ser758), and increased LC3B proteins. At the same time, suppressed expression of osteogenic markers (RUNX2, ALP, COL1A1, OSX) and alkaline phosphatase in response to AFF4 depletion were restored by rapamycin. Briefly, our results demonstrate that autophagy mediates regulatory function of AFF4 on osteogenic commitment of hPDLSCs.

**FIGURE 6:**
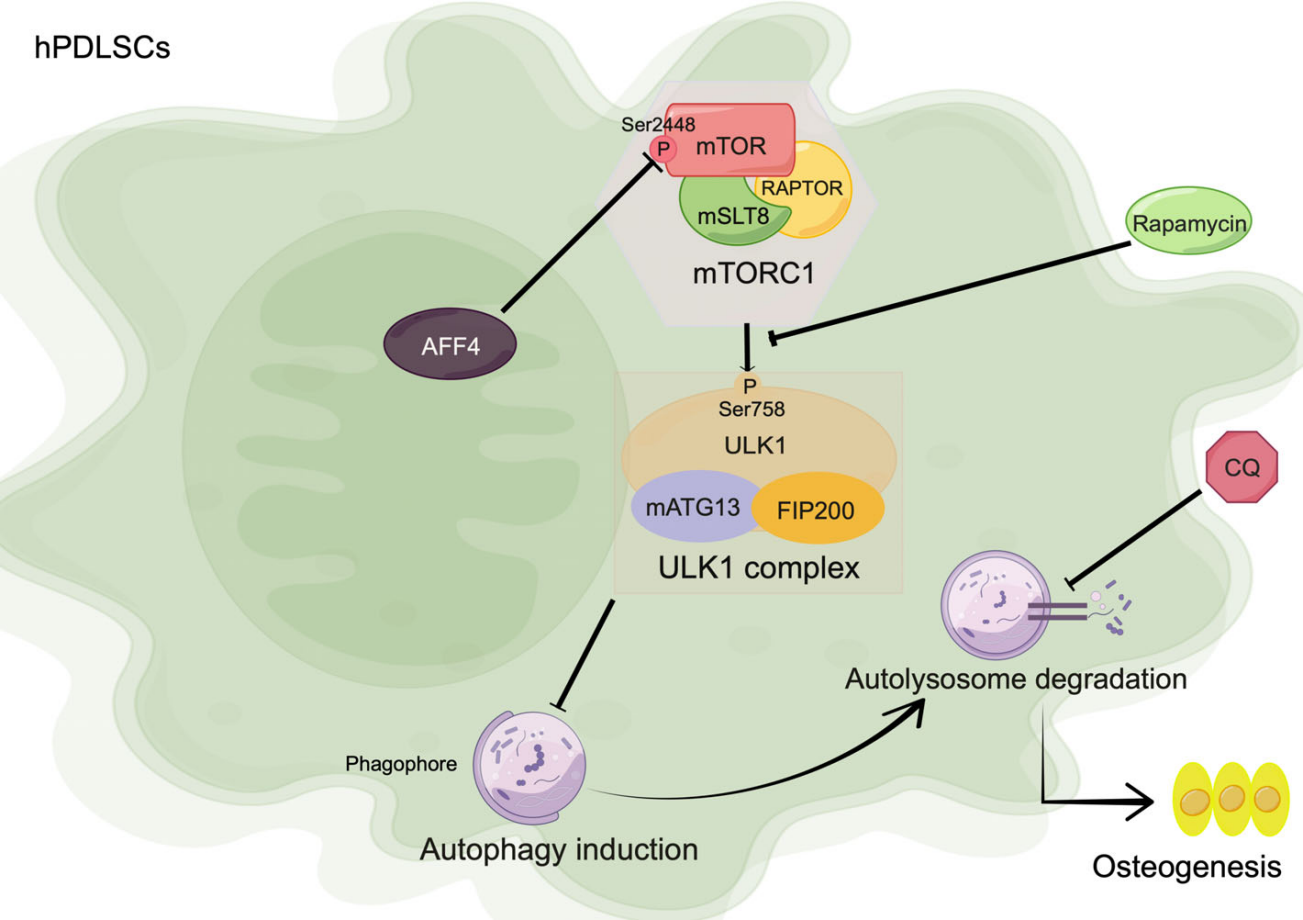
Schematic diagram of mechanisms underlying AFF4‐regulated osteogenic differentiation of hPDLSCs. (By Figdraw). AFF4 deficiency contributes to mTORC1 activation via phosphorylation of Ser2448, which subsequently inactivates ULK1 complex by phosphorylating Ser758 (human). Since ULK1 complex is essential for autophagy activation, suppressed autophagic activity finally leads to compromised osteogenic potential of hPDLSCs.

In summary, AFF4 is the scaffold protein of SEC, a complex required for rapid gene transcription in condition of development and stress. Meanwhile, periodontal inflammation is indeed a critical stress stimulus. Reduced expression of AFF4 in PDL tissues of periodontitis implicates that AFF4 might participate in periodontal dyshomeostasis in the condition of periodontitis. In our study, function of AFF4 in hPDLSCs was investigated for the first time. We discovered that AFF4 modulates osteogenic differentiation of hPDLSCs via targeting mTOR/ULK1/autophagy pathway (Figure 6). Therefore, our present study offers an important clue for in‐depth understanding of periodontitis niche, and also provide a novel and prospective target for possible gene therapy of periodontitis in future.

## AUTHOR CONTRIBUTIONS

Shujuan Zou, Chenchen Zhou, Zuping Wu and Li Zhu designed this study. Li Zhu, Sirui Chen and Yuying He conducted the experiment and collected the data. Li Zhu, Zuping Wu, Jiahe Wang and Guowen Luo performed data analysis and interpretation. Zhuoxuan Wu, Yuyu Li and Jing Xie checked all the data. Li Zhu drafted the manuscript. All author reviewed the manuscript content. Final approval was made by Shujuan Zou and Chenchen Zhou.

## CONFLICT OF INTEREST STATEMENT

The authors declare no conflicts of interest.

## Supporting information


**Data S1.** Supporting Information

## Data Availability

All original data that support the findings of this work are available from the corresponding author upon reasonable request.
