# Supplementary material for: AFF4 regulates osteogenic potential of human periodontal ligament stem cells via mTOR‐ULK1‐autophagy axis
Source: Cell Prolif. 2023 Sep 20;57(2):e13546. doi: 10.1111/cpr.13546 (PMC10849782; doi:10.1111/cpr.13546)
Supplement: Supplementary file 1 — Data S1. Supporting Information [file CPR-57-e13546-s001.docx]

**Table S1 Primer sequences for AF4/FMR2 family members and osteogenic genes**

| **Gene name** | **Gene ID** | **Primer sequences** |
| --- | --- | --- |
| **GAPDH** | NM_001256799.3 | Forward: AATGGGCAGCCGTTAGGAAA  Reverse: GCGCCCAATACGACCAAATC |
| **AFF1** | NM_001166693.3 | Forward: CTTTCCCACCGCAACAGGTC  Reverse: ACCCTTCCGTCCTCTAGTCC |
| **AFF2** | NM_001169122.2 | Forward: CACCTTTTCCATCCCAGGACA  Reverse: GACACTGACTTTGTAGAAGCTCTGG |
| **AFF3** | NM_001025108.2 | Forward: ACAGCTTCGACTTAGCCCTG  Reverse: GGTTGGAGAGTTCATCCCCC |
| **AFF4** | NM_014423.4 | Forward: ACTGGAGCAACATGAACCGT  Reverse: AGAGAGGAGAGCTAGGTGGG |
| **RUNX2** | NM_001015051.4 | Forward: ATCTCCGCAGGTCACTACCA  Reverse: ACTGTGCTGAAGAGGCTGTTT |
| **ALPL** | NM_000478.6 | Forward: ACCACCACGAGAGTGAACCA  Reverse: CGTTGTCTGAGTACCAGTCCC |
| **SP7 (OSX)** | NM_001173467.3 | Forward: GCCAGAAGCTGTGAAACCTC  Reverse: TGATGGGGTCATGGTGTCTA |

**Figure S1: Phenotypic identification of hPDLSCs**

**
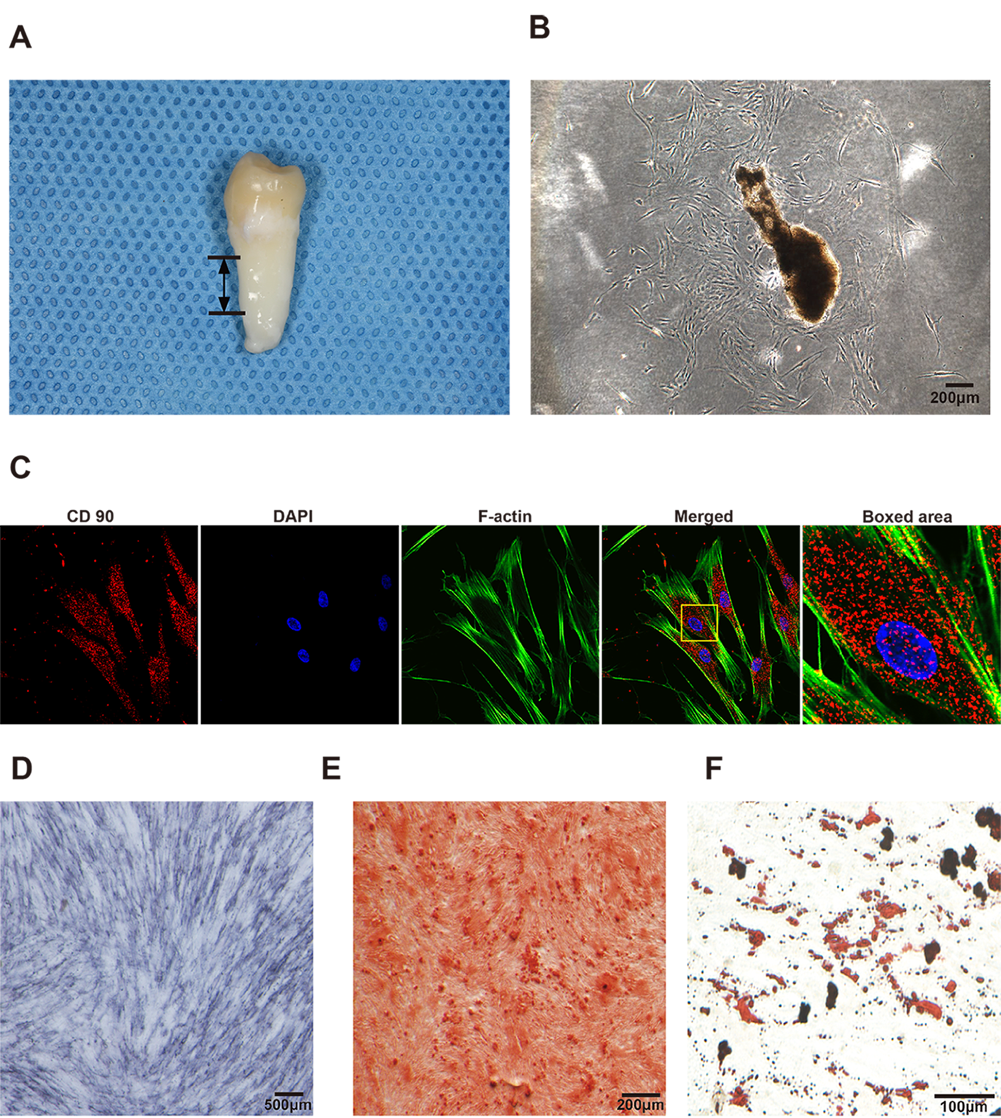
**

**Figure S1: Phenotypic identification of hPDLSCs.** (A) Representative images of tooth for hPDLSCs extraction. PDL tissues on the middle third of root were used. (B) Images of isolated hPDLSCs. (C) Expression of CD 90, surface marker of stem cells, were detected on hPDLSCs via IF. Multi-directional differentiation of hPDLSCs were identified via (D) ALP staining; (E) ARS staining; (F) Oil Red O staining.

**Figure S2: AFF4 knockdown efficiency at different time points**


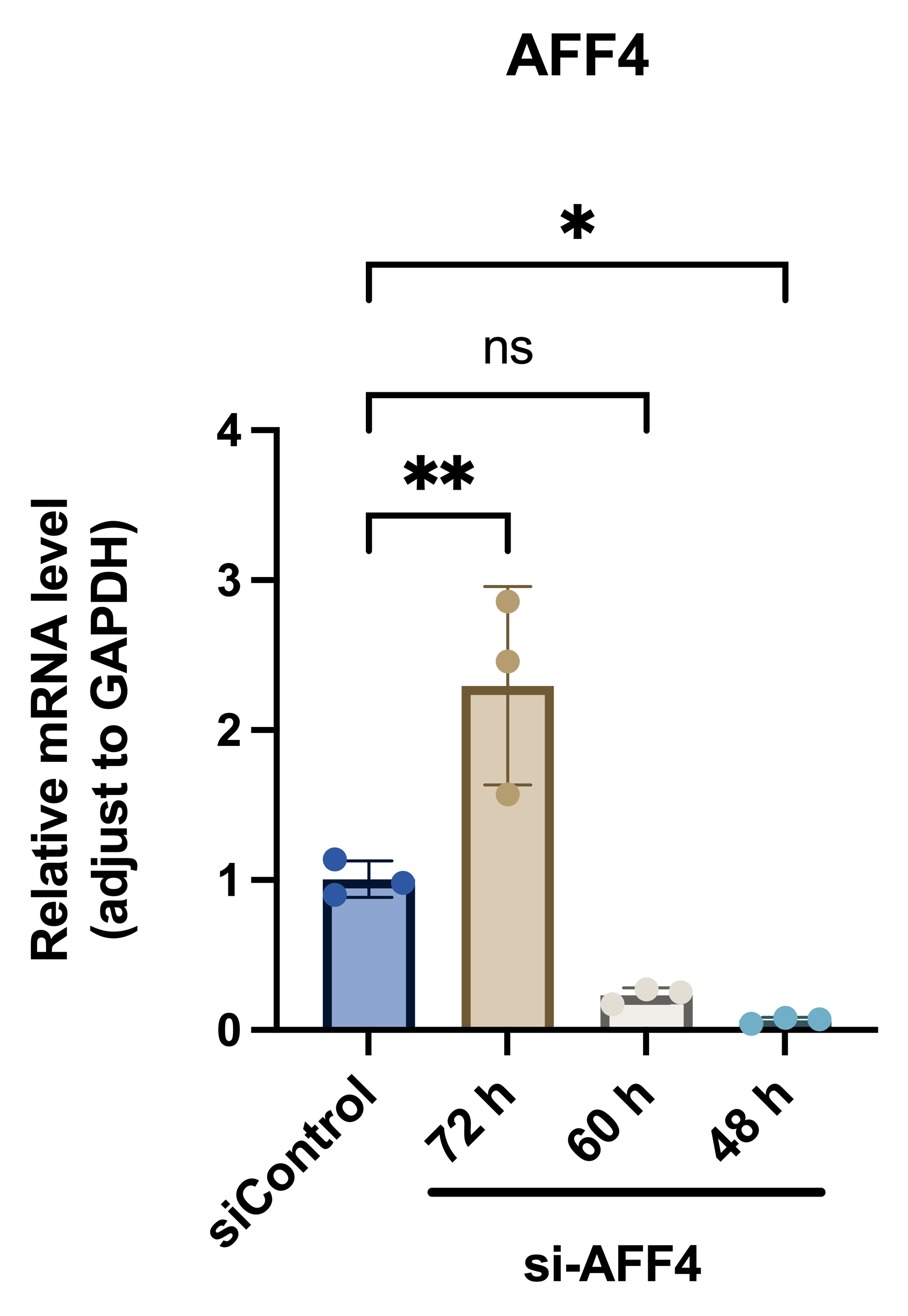


**Figure S3: Cell proliferative activity in condition of AFF4 knockdown**

**
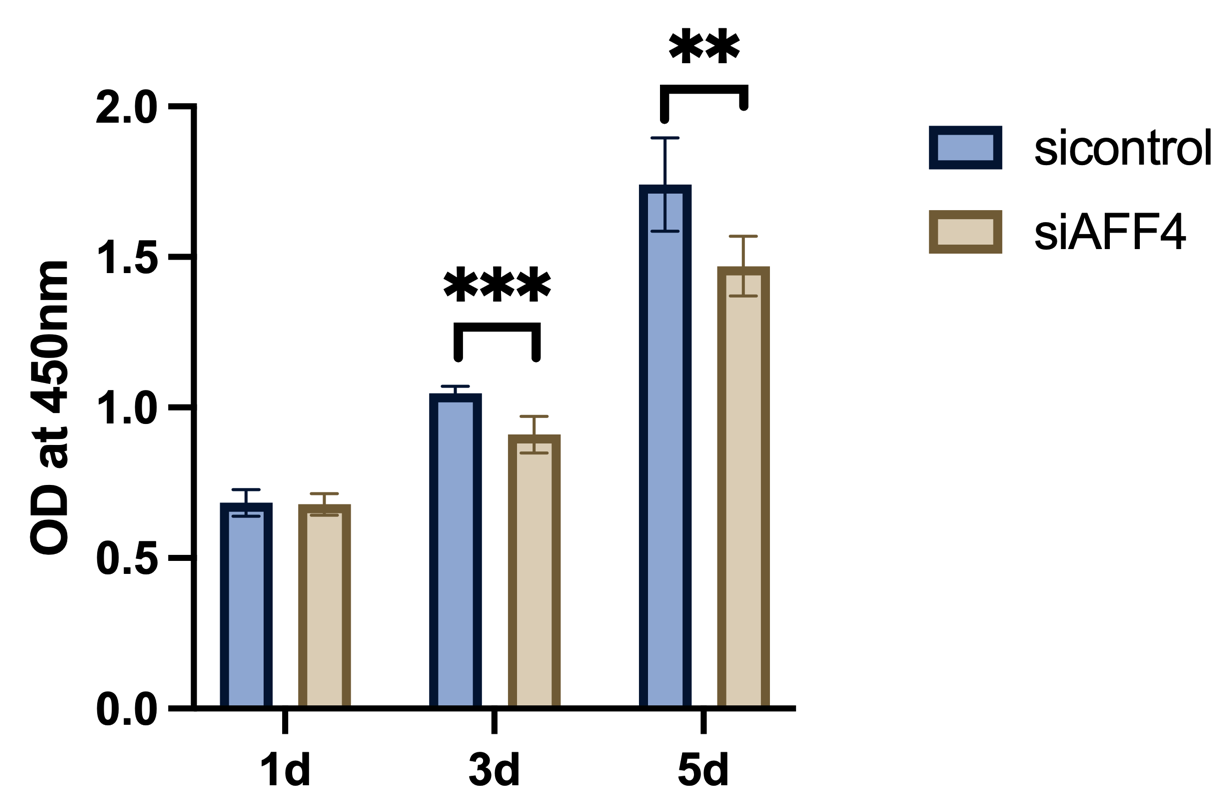
**

**Figure S4: Cytotoxity of LPS on hPDLSCs**

**
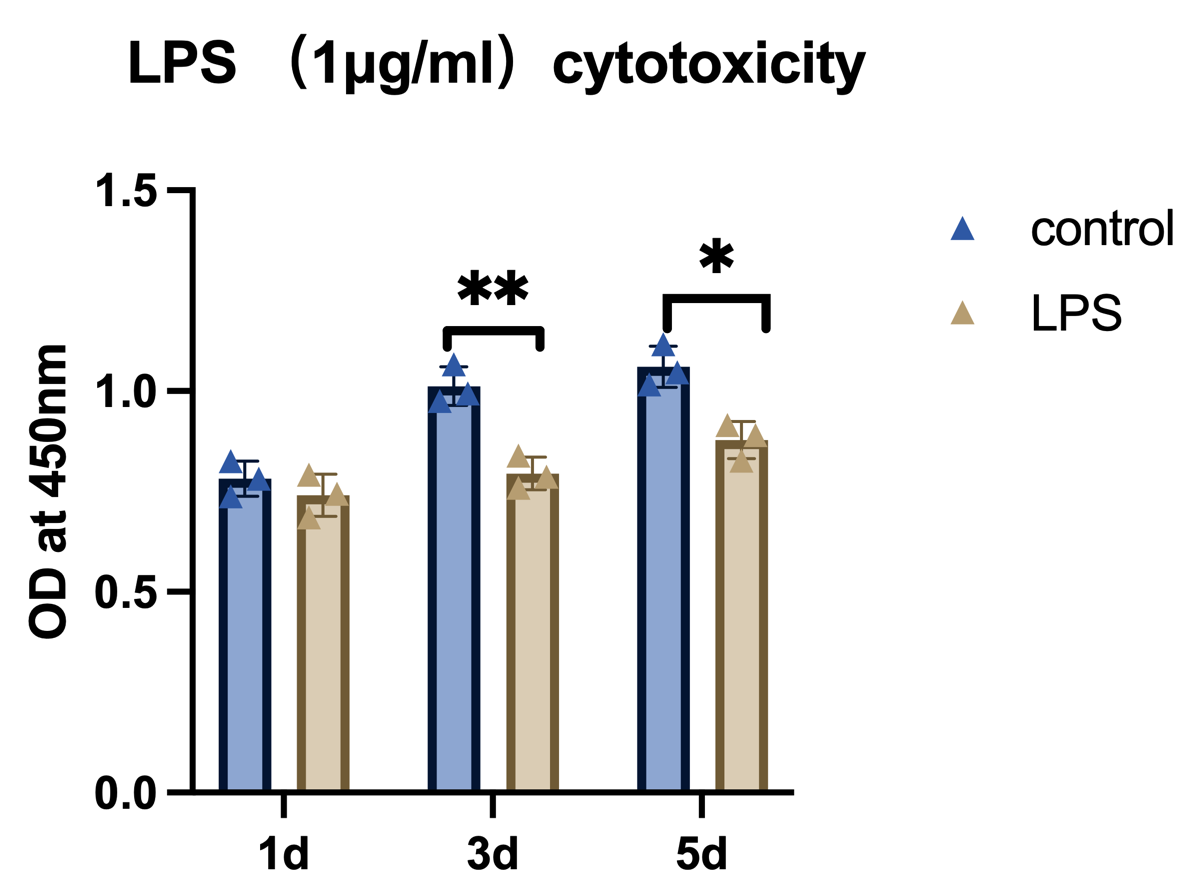
**
